# Supplementary material for: ASPPs multimerize protein phosphatase 1
Source: bioRxiv. 2025 May 19:2025.05.16.654433. Preprint. [Version 1] doi: 10.1101/2025.05.16.654433 (PMC12139778; doi:10.1101/2025.05.16.654433)
Supplement: 1 [file NIHPP2025.05.16.654433v1-supplement-1.pdf]

bioRxiv preprint doi: https://doi.org/10.1101/2025.05.16.654433; this version posted May 19, 2025. The copyright holder for this preprint (which was not certified by peer review) is the author/funder, who has granted bioRxiv a license to display the preprint in perpetuity. It is made available under aCC-BY 4.0 International license.

Supporting information

S1 Fig. APE-1 function requires its N-terminal helix.

(A) Alphafold structural domain predictions mapped onto the primary sequences of ASPP1, ASPP2, and iASPP from H. sapiens as well as APE-1 from C. elegans. Predicted structures of the N-terminal alpha helices are shown in the insets above each

bioRxiv preprint doi: https://doi.org/10.1101/2025.05.16.654433; this version posted May 19, 2025. The copyright holder for this preprint (which was not certified by peer review) is the author/funder, who has granted bioRxiv a license to display the preprint in perpetuity. It is made available under aCC-BY 4.0 International license.

domain map with predicted residues numbers indicated. Represented in different colors are the beta-grasp domain (teal), N-terminal alpha helix (tan), undefined alpha-helical regions (brown), ankyrin repeats (purple), and SH3 domain (magenta). (B) Body length assay. Data represent mean and S.E.M. (black bars) of 23-29 biological replicates.

S4 Fig. Convolutional Neural Network (CNN) confusion matrices.

(A) CNN-Green mean confusion matrix of raw counts averaged across 5-fold cross validation during training with GFP fluorescence intensity traces. (B) Row-normalized mean confusion matrix from panel A showing recall for each class during K-fold cross validation of the CNN-Green. (C) CNN-FarRed mean confusion matrix of raw counts averaged across 5-fold cross validation during training with far-red fluorescence intensity traces. (D) Row-normalized mean confusion matrix from panel C showing recall for each class during K-fold cross validation of the CNN-FarRed.

S5 Fig. GSP-2 oligomers bypass APE-1 missense mutations

(A) Body length assay.Data represent the mean and S.E.M. (black bars) of 27-36 biological replicates. **** indicates $p<0.0001$. * indicates $p<0.05$.

S1 File. Training metrics from CNN-Green K-fold cross validation.

S2 File. Training metrics from CNN-FarRed K-fold cross validation.

S3 File. Strains, alleles, and reagents.
